# Outcome Measures in Multiple Sclerosis

**DOI:** 10.1155/2014/439375

**Published:** 2014-05-05

**Authors:** Robert Bermel, Amy Waldman, Ellen M. Mowry

**Affiliations:** ^1^Cleveland Clinic, Cleveland, OH 44195, USA; ^2^University of Pennsylvania School of Medicine, Philadelphia, PA 19104, USA; ^3^Johns Hopkins University School of Medicine, Baltimore, MD 21287, USA

The acquisition of a reliable and valid outcome measure in multiple sclerosis (MS) is one of the holy grails for clinician researchers working in the field. The current standard for measuring MS-related disability is the Expanded Disability Status Scale (EDSS), a nonlinear scale ranging from 0 (normal neurologic examination) to 10 (death due to MS) [1]. Widely incorporated into MS clinical trials, the EDSS has provided a critical framework for quantifying the toll that MS takes on those who suffer from it and has been considered the “gold standard” for measuring MS-related disability. Because the science to do so was not advanced enough at the time the EDSS was initially developed, however, its measurement properties were never properly evaluated at the time of its creation [2].

Practitioners who see patients with MS can attest to limitations in the neurologic examination, which essentially serves as the basis for the EDSS, in capturing the current clinical status of a patient. For example, a patient diagnosed by one of the editors with primary progressive MS who had an initial EDSS score of 2.0 due to foot drop returned a few months later with resolution of the foot drop (EDSS 1.0), which brought into question whether the course was truly progressive. However, the patient reported continued progression in difficulty functioning, noting that the disease had eliminated the capacity to jog, a favorite pastime. Further, when confronted with the puzzling improvement in the neurologic exam, the patient revealed that while at the previous visit, they had arrived at the office exhausted after walking several miles to get there; they had been dropped off in front of the clinical building for the follow-up visit.

This story illustrates some of the problems with the use of the EDSS as a solitary outcome measure in MS. First, it brings the reliability of the scale into question. That a person with MS, particularly with a progressive course, could have such marked fluctuations and indeed even improvements in the score makes the interpretation of the scores difficult. While the EDSS has been shown to be a valid measure of physical disability in adults, there are problems with its reliability, capacity to discriminate between patients or groups thereof, and responsiveness [2]. Further, while the patient in this illustrative case reported worsening physical functioning that greatly impacted subjective quality of life at the second visit, the EDSS score was 1.0, indicating minimal disability. By design, the EDSS was not intended to be a comprehensive measure of the total impact MS has on the life of someone with the disease [2]. These limitations have prompted the search for better outcome measures for use by regulatory agencies approving new medications and for clinicians caring for patients with MS [3].

In this special edition of Multiple Sclerosis International, we sought to include papers that focused on outcome measures for MS. The articles herein range in scope from evaluations of the measurement properties of currently employed definitions of “sustained progression” using the EDSS to the development of a new relapse assessment measure and to measuring specific aspects of MS-related problems, including imbalance, walking impairment, and reduced quality of life. Studies of paraclinical outcomes, including optical coherence tomography and diffusion tensor imaging, and their relation to disability are also included.

The reader will appreciate that no single outcome measure is likely to fulfill all needs. Clinical measures that most closely capture the patient's current level of performance or functioning are subject to fluctuations and may be temporally or physiologically disconnected from the disease pathology targeted by treatment. Imaging outcomes may capture features that are more proximate to the pathophysiology of MS but may not reflect findings that will become clinically meaningful. The body of work featured here adds to the dialogue that will inform the development and refinement of outcome measures that more fully capture and quantify the patient experience of MS.

*Robert Bermel* *Robert Bermel* *Amy Waldman* *Amy Waldman* *Ellen M. Mowry* *Ellen M. Mowry*
